# ASIC3 plays a protective role in delayed-onset muscle soreness (DOMS) through muscle acid sensation during exercise

**DOI:** 10.3389/fpain.2023.1215197

**Published:** 2023-09-19

**Authors:** Tahsin Khataei, Christopher J. Benson

**Affiliations:** ^1^ Department of Internal Medicine, Roy J and Lucile A. Carver College of Medicine, University of Iowa, Iowa City, IA, United States; ^2^ Iowa City VA Healthcare System, Iowa City, IA, United States; ^3^ Department of Health and Human Physiology, University of Iowa, Iowa City, IA, United States

**Keywords:** ASICs, muscle afferent, sensory neuron, exercise, pain, delayed-onset muscle soreness, acidosis, lactate

## Abstract

Immediate exercise-induced pain (IEIP) and DOMS are two types of exercise-induced muscle pain and can act as barriers to exercise. The burning sensation of IEIP occurs during and immediately after intensive exercise, whereas the soreness of DOMS occurs later. Acid-sensing ion channels (ASICs) within muscle afferents are activated by H^+^ and other chemicals and have been shown to play a role in various chronic muscle pain conditions. Here, we further defined the role of ASICs in IEIP, and also tested if ASIC3 is required for DOMS. After undergoing exhaustive treadmill exercise, exercise-induced muscle pain was assessed in wild-type (WT) and *ASIC3*^−/−^ mice at baseline via muscle withdrawal threshold (MWT), immediately, and 24 h after exercise. Locomotor movement, grip strength, and repeat exercise performance were tested at baseline and 24 h after exercise to evaluate DOMS. We found that *ASIC3*^−/−^ had similar baseline muscle pain, locomotor activity, grip strength, and exercise performance as WT mice. WT showed diminished MWT immediately after exercise indicating they developed IEIP, but *ASIC3*^−/−^ mice did not. At 24 h after baseline exercise, both *ASIC3*^−/−^ and WT had similarly lower MWT and grip strength, however, *ASIC3*^−/−^ displayed significantly lower locomotor activity and repeat exercise performance at 24 h time points compared to WT. In addition, *ASIC3*^−/−^ mice had higher muscle injury as measured by serum lactate dehydrogenase and creatine kinase levels at 24 h after exercise. These results show that ASIC3 is required for IEIP, but not DOMS, and in fact might play a protective role to prevent muscle injury associated with strenuous exercise.

## 1. Introduction

Exercise is increasingly considered as a non-pharmacological, low cost and readily available first-line therapy for many chronic disease conditions (1–4), as well as to prevent the development of chronic pain (5). However, despite the many benefits of regular physical activity, exercise can induce pain and fatigue (6). These negative responses to exercise can be barriers to participating in regular physical activity, especially in individuals already suffering from chronic pain and fatigue conditions.

Immediate exercise-induced pain (IEIP) and delayed onset muscle soreness (DOMS) are two types of muscle pain induced by exercise. IEIP is generally described as a burning sensation usually experienced during and immediately after intense exercise and is believed to be caused by the accumulation of metabolites (e.g., H^+^, lactate, and ATP) within the exercising muscle. DOMS, on the other hand, is an aching muscle pain and discomfort that peaks between 24 and 48 h after exercise and is caused by excessive and unaccustomed muscle contractions. Besides muscle soreness, DOMS is also characterized by stiffness, weakness, tenderness, and diminished movement (7–10).

While the mechanisms underlying DOMS are incompletely understood, the most accepted purported causes involve reversible microscopic damage and associated inflammation of muscle fibers (7, 9, 11–13). These micro-injuries lead to the recruitment of neutrophils and macrophages that release several inflammatory and growth factors. The accumulation of metabolites during and immediately after exercise, and the increase in inflammatory mediators and neurotrophic factors occurring 1–2 days later, are sensed by receptors on muscle sensory (afferent) neurons, which are in turn activated and signal the sensations of IEIP and DOMS. A likely candidate sensor of these chemical perturbations within muscle afferents are acid-sensing ion channels (ASICs) (12, 14–17).

ASICs are H^+^-gated ion channels of the DEG/ENaC family principally expressed in central and peripheral sensory neurons (18, 19). They include four genes (*ASIC1*, -*2*, -*3*, and -*4*) that encode for six subunits (*ASIC1* and -*2* both have alternative splice transcripts: ASIC1a, -1b, -2a, and -2b) (20–22). Functional ASIC channels consist of a complex of three subunits; individually expressed subunits form homomultimeric channels, whereas coexpression of two or more subunits generates heteromultimeric channels (23). They are highly expressed in skeletal muscle afferents (18, 24), where they play several important functions. First, ASICs are poised to sense chemical changes with skeletal muscle—they are activated in the range of extracellular pH (7.0–6.8) that occurs during muscle ischemia and exercise, and are potentiated by other chemicals during muscle ischemia, hypoxia, and/or exercise (e.g., lactate, ATP, arachidonic acid, and nitric oxide) (14–17, 25, 26). We studied isolated labeled muscle afferents from mice and found that ∼70% displayed ASIC-like currents, and the subunit composition was a heteromeric channel consisting primarily of ASIC1a, -2a, and -3 subunits (27). Second, ASICs are required for the development of chronic muscle pain. Either genetic deletion, RNAi knockdown in muscle afferents, or pharmacological inhibition of ASICs attenuates hyperalgesia in rodent models of chronic muscle pain (28–31). Third, ASICs play an important role in muscle afferents during intense exercise. Type III (thinly myelinated) and type IV (unmyelinated) muscle afferents are activated by both mechanical and/or metabolic (chemical) stimuli during exercise, which triggers reflexive increases in BP, HR, and ventilation via sympathetic nerve activity (termed the “exercise pressor reflex”) (29, 32, 33). In a series of studies, pharmacological or genetic inhibition of ASICs have been shown to attenuate the metabolic component, but not the mechanical component, of the exercise pressor reflex (34–39). Additionally, we recently showed that ASICs are required for IEIP—genetic deletion of *ASIC3* abolished muscle hyperalgesia immediately after exhaustive exercise (40). Additionally, exercise training downregulated the expression of ASICs in muscle afferents, and was associated with lower IEIP and improved exercise performance after training (40).

There is also evidence that ASICs might play a role in a rat model of DOMS. Fuji et al. found that intraperitoneal or intramuscular injection of an ASICs antagonist (amiloride) prevented muscle hyperalgesia induced by electrically stimulated eccentric muscle contraction (41). More recently, this same group found that intramuscular injection of APETx2, a selective blocker of ASIC3, similarly prevented muscle hypersensitivity and suppressed the hyperexcitability of isolated muscle afferent fibers after eccentric contraction (42).

While acknowledging the above data supporting a role for ASICs in DOMS, we also reasoned that ASICs could have an opposite effect and might actually serve a protective role to attenuate DOMS. While exercise performance can be limited by the sensations of muscle pain (43, 44), acute pain can also be protective against harm and muscle injury (45–47). We hypothesized that IEIP could serve as an alarm to prevent over-exercising and given that *ASIC3*^−/−^ mice do not sense IEIP they might experience more muscle fatigue and potentially more injury during exhaustive exercise. Additionally, loss of normal increases in blood pressure, heart rate, and respiration via the exercise pressor reflex in *ASIC3*^−/−^ mice might induce more physiological stress on the muscle during exercise. Thus, the current study was conducted to (1) further our understanding of the role of ASICs on muscle pain immediately after exhaustive exercise, and (2) to investigate the role of ASICs in DOMS using a genetic knockout model of ASIC3. We chose to study ASIC3 because it is not expressed in the central nervous system in mice (only expressed in peripheral sensory neurons), and it plays a large role in the biophysical properties of ASIC currents in mouse muscle afferents (27).

## 2. Methods

### 2.1. Ethical approval

All experimental procedures and protocols were approved by the Institutional Animal Care and Use Committee of the University of Iowa (Protocol no. 1990903) and conformed to the national guidelines set by the Association for Assessment and Accreditation of Laboratory Animal Care. All experiments were carried out according to the guidelines described by Grundy (48), and we have taken all steps to minimize the pain and suffering of the animals.

### 2.2. Animals

Male and female 11–12-week-old C57BL/6J (Jackson Laboratory, Sacramento, CA, USA) or *ASIC3*^−/−^ mice were acclimated to the new environment and room for 1 week prior to the beginning of treadmill acclimation. The generation of *ASIC3*^−/−^ mice has been previously reported (49). These mice were subsequently backcrossed for 10 generations onto a C57BL/6J background to generate a congenic line. The animals were housed in a temperature-controlled room (22°C) with a 12-hour light/dark cycle and had free access to standard mouse food (pellet) and water *ad libitum*. Mice were euthanized with CO_2_ per AVMA guidelines. All tests were performed by the same experimenter who was blinded to genotype and drug injection in all experiments. All experiments were performed in both male and female mice. We found no differences between sexes and so they were combined in all experiments.

### 2.3. Exhaustive exercise protocol

Mice were acclimated to a six-lane motor-driven treadmill (Columbus Instruments, Columbus, OH) for 3 days with gradually increasing velocity and incline for 30 min/day. Shock grids at the rear of the treadmill were set at 1 mA and 1 Hz to encourage continued ambulation on the treadmill during acclimation. Exhaustive exercise testing was performed to measure time to exhaustion as previously described (40). Briefly, mice were placed on a non-moving treadmill (20° incline throughout) with a shock bar on for 10 min, followed by a 10-min warm-up at a velocity of 6 m/min. At the beginning of exhaustive exercise testing, we turned off the shock bar and instead used gentle prodding with a tongue depressor to encourage exercise as needed. We found this technique to be less stressful for the mice, and exhaustive exercise capacity was similar compared to using the shock bars (50). After the warm-up, the velocity was increased to 8 m/min, and then increased by 2 m/min every 3 min (velocity accelerations were 2 m/min^2^) until the mice became exhausted. Exhaustion was defined as the point at which mice stayed at the end of treadmill and did not respond to 10 consecutive nudges with a tongue depressor in 10 s.

### 2.4. Open field test

For the assessment of locomotor activity, mice were subjected to the open field test before, immediately and 24 h following exercise. Horizontal movements were quantified as the number of photobeam breaks over 30 min in a lighted chamber (40.6 cm wide × 40.6 cm deep × 36.8 cm high, 1,500 lux, 16 × 16 photobeams to record horizontal movements, with 2.54 cm photobeam spacing) (San Diego Instruments).

### 2.5. Muscle withdrawal threshold (MWT)

MWT was measured by applying a force-sensitive tweezer to the belly of the gastrocnemius muscle as previously described (51), whereby lower thresholds indicate greater sensitivity to mechanical stimuli. Briefly, mice were acclimated to the testing paradigm in two 5-min sessions for 3 days. Then, mice were gently restrained in a gardener's glove, the hindlimb held in extension, and the muscle squeezed with progressive force with the force-sensitive tweezers until the mouse withdrew its hindlimb. Three trials per hindlimb were averaged. In this study, we selected the gastrocnemius muscle because it is the main muscle during running on the treadmill in mice and the fast twitch muscles are more susceptible to damage after exercise (12, 52).

### 2.6. Grip strength test

Limb grip strength was measured using a grip strength meter (Bioseb, Model: Bio-GT3, France). Mice were acclimated to the apparatus by being placed on the grids twice per day for 5 s in 1 min intervals for 3 consecutive days. Grip strength was then measured by placing mice on the grid and then gently pulling their tail (at a constant speed within 3 s) to measure maximal grip force (calculated by averaging 3 trials conducted at 1 min intervals). The grip test was performed at baseline and 24 h after exercise.

### 2.7. Lactate measurement

Blood lactate was measured using the tail nick technique to collect 0.5 μl of blood and analyzed via a lactate meter (Nova Biomedical, Waltham, MA) (53).

### 2.8. Serum CK and LDH assessments

Mice were anesthetized under 2% isoflurane and blood was collected via cardiac puncture. Samples were transferred into a serum collection tube, allowed the blood to clot at room temperature for 20 min, then centrifuged at 3,000 *g* for 15 min. Frozen serum was shipped to IDEXX BioAnalytics Labs in Sacramento, CA. Serum samples were run using the Beckman Coulter AU680 automated chemistry platform and Beckman Coulter reagents. All chemistry assays are validated for a serum matrix. The analyzer performs photometric and potentiometric assays. Creatine kinase (CK) and lactate dehydrogenase (LDH) are both kinetic enzymatic assays read spectrophotometrically.

### 2.9. Statistical analysis

GraphPad Prism (8.0) was used to statistically analyze data. Bar graphs represent means ± standard error of the mean (SEM). One-way or two-way ANOVA, or *t*-tests were used to analyze the results as described in figure legends. Paired or repeated measures testing was done if mice were tested sequentially. If significant differences were observed, then the indicated *post hoc* tests were performed. Values of *P* < 0.05 were considered statistically significant. Sample sizes for each experimental protocol were determined based on the effect sizes for changes in the variable from our previous and pilot studies (*α* = 0.05, *β* = 0.8; G*Power 3.1 software (54).

### 2.10. Experimental design

**Figure 1:**
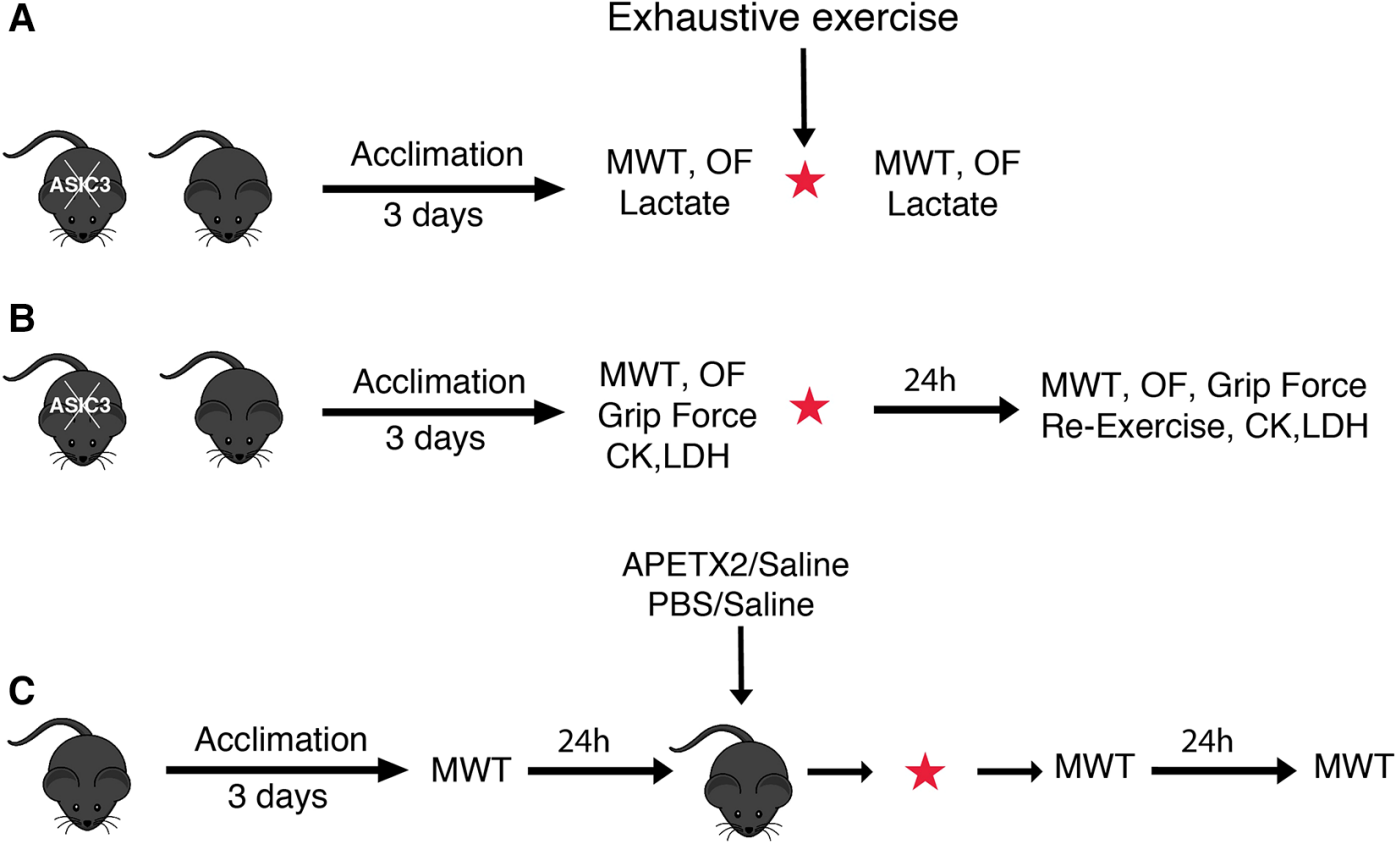
Schematic illustrations of experimental designs to test IEIP (**A**), DOMS (**B**), and muscle injection protocols (**C**). Separate groups of mice were used for each experiment. Additionally, separate groups of mice were used pre- and post-exercise for OF and CK, LDH experiments. MWT, muscle withdrawal threshold test; OF, open field test; CK, creatine kinase; LDH, lactate dehydrogenase; APETX2, a selective and reversible ASIC3 inhibitor; PBS, phosphate buffered saline.

To test for IEIP, separate groups of mice underwent 3 days of treadmill acclimation prior to exhaustive exercise testing (exercise group) or were placed on a non-moving treadmill for the same amount of time (control group). Exercise-induced muscle pain (using MWT), locomotor activity (using open field test), or blood lactate levels were assessed at baseline (a day before exercise), and immediately after exercise (Figure 1A). To test for DOMS (Figure 1B), we acclimated separate groups of mice and performed exhaustive exercise as above, and measured MWT, open field test, grip force, and serum CK and LDH before (baseline) and 24 h after exercise [the peak of DOMS has been observed 12–24 h after exercise in mice (55)]. We also performed the same exhaustive running exercise protocol 24 h after the primary exercise test (re-exercising) (Figure 1B). Each experiment was performed in separate groups and in both male and female WT and *ASIC3*^−/−^ mice. We found no differences between male and female mice and so their data is combined. For open field and CK, LDH experiments, separate groups of mice were used at baseline and after exercise due to learning effects (for open field test) and blood collection technique (for CK, LDH assessment), whereas for all other assays mice were tested before and after exercise.

In separate studies we injected the ASIC3 inhibitory toxin APETx2 [20 μl; 200 nM in 0.9% sodium chloride (saline)] or phosphate buffered saline [PBS (Sigma, MO, US); 20 μl; 137 mM NaCl, 2.7 mM KCl, 10 mM Na_2_HPO_4_, and 1.8 mM KH_2_PO_4_, pH 7.4] into the right or left (randomly chosen) gastrocnemius muscle and saline (20 μl) as control into the contralateral muscle 5 min before the exhaustive exercise using a 0.5 ml syringe (31 gauge needle). Muscle withdrawal threshold (MWT) was assessed at baseline (a day before exercise), immediately, and 24 h after exercise (Figure 1C).

## 3. Results

### 3.1. ASIC3 is required for immediate exercise-induced muscle pain (IEIP)

**Figure 2:**
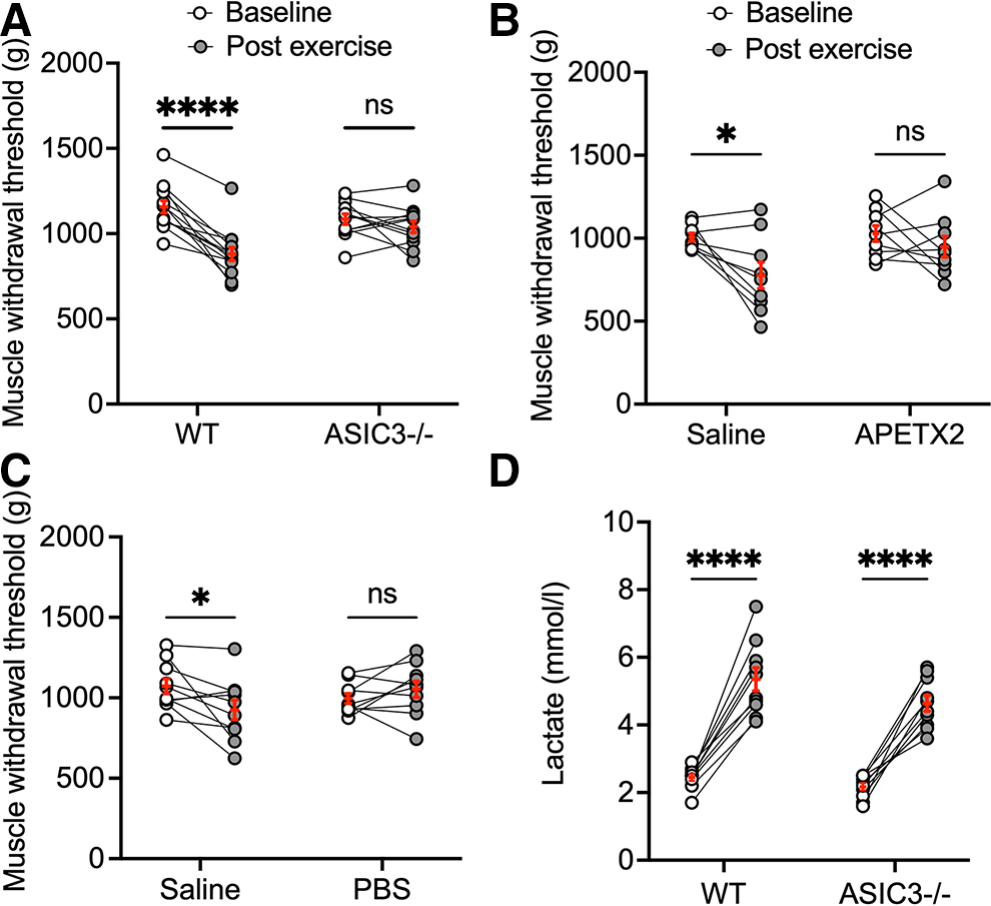
Effect of genetic deletion and pharmacological inhibition of ASIC3 on immediate exercise-induced pain (IEIP). (**A**) Muscle withdrawal threshold (MWT) was measured before (baseline) and immediately after exhaustive exercise in male and female wild-type (WT) and *ASIC3*^−/−^ mice. Two-way repeated measures ANOVA showed a significant time effect [*F*_(1,23)_ = 35.27, *P* < 0.0001, *n* ≥ 12]. Sidak's multiple comparison test found no difference in baseline MWT between groups before exhaustive exercise. On the other hand, there was a significant decrease in MWT immediately after exercise compared with baseline for WT (****P < 0.0001) but not *ASIC3*^−/−^ mice. Reproduced and modified from Khataei et al. 2020 (40). (**B**) Twenty microliter APETX2 (200 nM) was injected into the right or left (randomly chosen) gastrocnemius muscle and saline (20 μl) as control into the contralateral muscle 5 min before the exhaustive exercise. MWT was assessed at baseline and immediately after exhaustive exercise. Two-way repeated measure ANOVA showed a significant time effect [*F*_(1,16)_ = 8.25, *P* < 0.05, *n* = 9]. Sidak's multiple comparison test discovered MWT was diminished immediately after exercise with saline (*P* < 0.05) but not with APETX2. (**C**) Twenty microliter saline or Phosphate-Buffered Saline (PBS) was injected into contralateral gastrocnemius muscles 5 min before the exercise. MWT was assessed at baseline and immediately after exhaustive exercise. Two-way repeated measure ANOVA showed a significant group-time effect [*F*_(1,18)_ = 7.69, *P* < 0.05, *n* = 10]. Sidak's multiple comparison test discovered MWT was diminished immediately after exercise with saline (*P* < 0.05) but not with PBS. (**D**) Blood lactate was measured before and immediately after exhaustion. Two-way repeated measure ANOVA found a significant time effect [*F*_(1,18)_ = 144.9, *P* < 0.0001, *n* = 10]. Sidak's multiple comparison test showed a significant increase in blood lactate for both WT and *ASIC3*^−/−^ mice following exhaustive exercise (*P* < 0.0001).

We previously found that the genetic deletion of ASIC3 abolished muscle hyperalgesia immediately after exhaustive exercise (40). As a measure of muscle pain, we recorded MWT, whereby force-sensitive tweezers were applied to the gastrocnemius muscle until the limb was withdrawn (a lower MWT indicated increased pain or hyperalgesia) before and immediately after exhaustive exercise in *ASIC3*^−/−^ and WT mice. We found that there was no difference in MWT at baseline between *ASIC3*^−/−^ and WT. However, WT mice had a significantly lower MWT immediately after exercise indicating higher IEIP, whereas the *ASIC3*^−/−^ mice did not show any changes in MWT immediately after exercise indicating no changes in pain perception immediately after exercise (Figure 2A). To test if ASIC3 is required specifically within muscle afferents for IEIP, we injected a specific ASIC3 inhibitory peptide APETx2 (20 μl, 200 nM) into the right or left gastrocnemius muscle (randomly assigned), and a similar volume of saline (control) was injected into the contralateral muscle 5 min before the exercise test. Similar to the genetic deletion of ASIC3, the limb that was injected with saline developed lower MWT immediately after exercise, whereas the decrease in post-exercise MWT was abrogated in the limb that received APETx2 (Figure 2B).

Next, we tested if acidosis within the exercising muscle is required for IEIP. We found that PBS injected into the gastrocnemius muscle 5 min before exercise completely abolished the reduction in MWT after exercise, indicating buffering pH within exercising muscle was able to eliminate muscle hyperalgesia immediately after exercise (Figure 2C). We also tested lactate levels immediately after exercise to ascertain if this could account for the differences in pain perception immediately after exercise. Our results showed that both *ASIC3*^−/−^ and WT mice had significant increases in serum lactate compared to their baselines, and there was no difference between the two groups (Figure 2D). In summary, these results demonstrate that ASIC3 within muscle afferents is required for muscle hyperalgesia immediately after exhaustive exercise, and that acidosis is a necessary mediator of this increase in pain perception.

### 3.2. While *ASIC3*^−/−^ mice have similar exercise capacity, they have increased fatigue immediately after exercise

**Figure 3:**
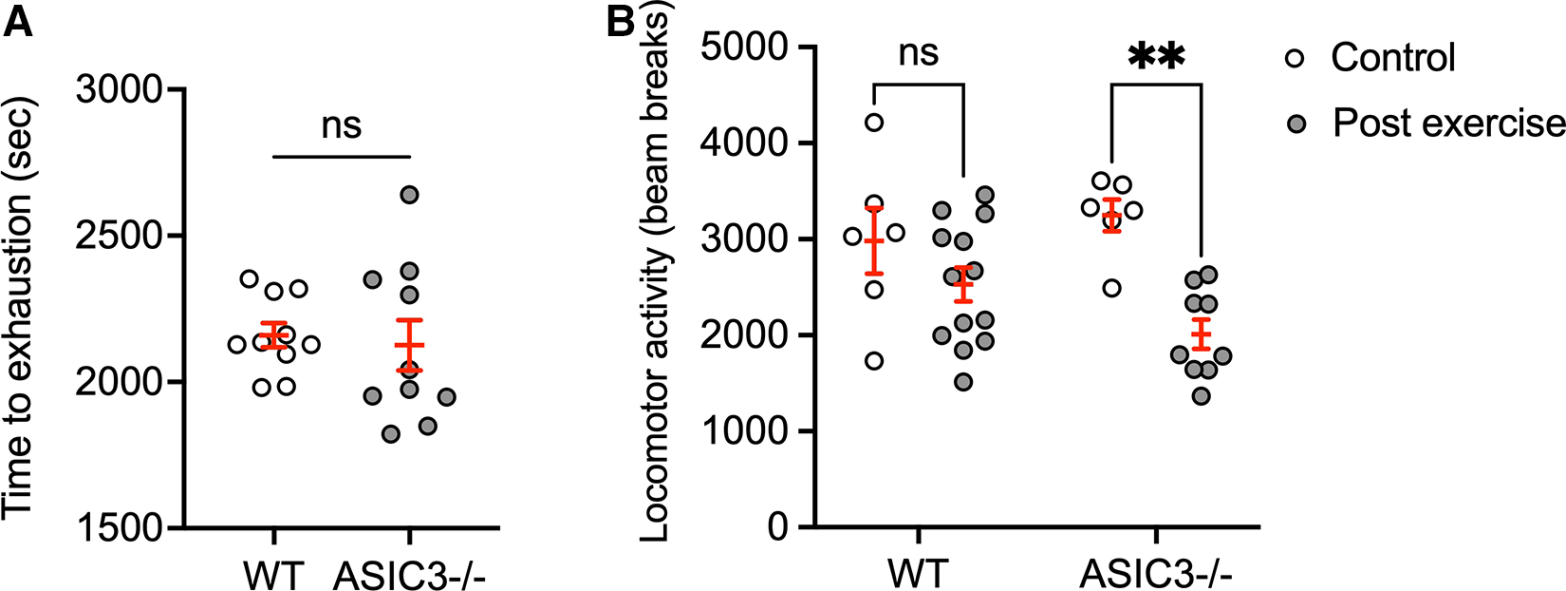
Effect of genetic deletion of *ASIC3* on exercise capacity (**A**) and locomotor activity immediately after exercise (**B**). (**A**) Time to exhaustion (sec) was measured during exhaustive exercise test. Unpaired Student's *t*-test revealed no difference between groups. (**B**) Locomotor activity (beam breaks) in open field assay measured for 30 min immediately after exhaustive exercise or mice placed on a non-moving treadmill (control). Two-way ANOVA showed a significant time effect [*F*_(1,30)_ = 15.11, *P* < 0.001, *n* ≥ 6]. Sidak's multiple comparison test revealed significantly lower total beam breaks for *ASIC3*^−/−^ post exercise compared to control *ASIC3*^−/−^ mice (*P* = 0.001) but no difference for WT mice (ns).

Since *ASIC3*^−/−^ mice had diminished muscle hyperalgesia immediately after exercise, we hypothesized that they might have higher exercise performance and increased fatigue immediately after exercise. Interestingly, we found *ASIC3*^−/−^ mice had a similar time to exhaustion on treadmill testing compared to WT mice (Figure 3A). To test for fatigue, we recorded locomotor activity for 30 min in an open field assay immediately after exhaustive exercise. Figure 3B shows that WT mice had similar locomotor activity after exercise compared to WT mice that were placed on a non-moving treadmill. While there was not a statistical difference between genotypes post-exercise, *ASIC3*^−/−^ mice had a significantly lower activity immediately after exercise compared to *ASIC3*^−/−^ mice that did not exercise (Figure 3B). Thus, while *ASIC3*^−/−^ mice had similar exercise capacity as WT mice, our data suggests they have increased fatigue and delayed recovery after exercise.

### 3.3. *ASIC3*^−/−^ mice experience increased DOMS compared to WT mice

**Figure 4:**
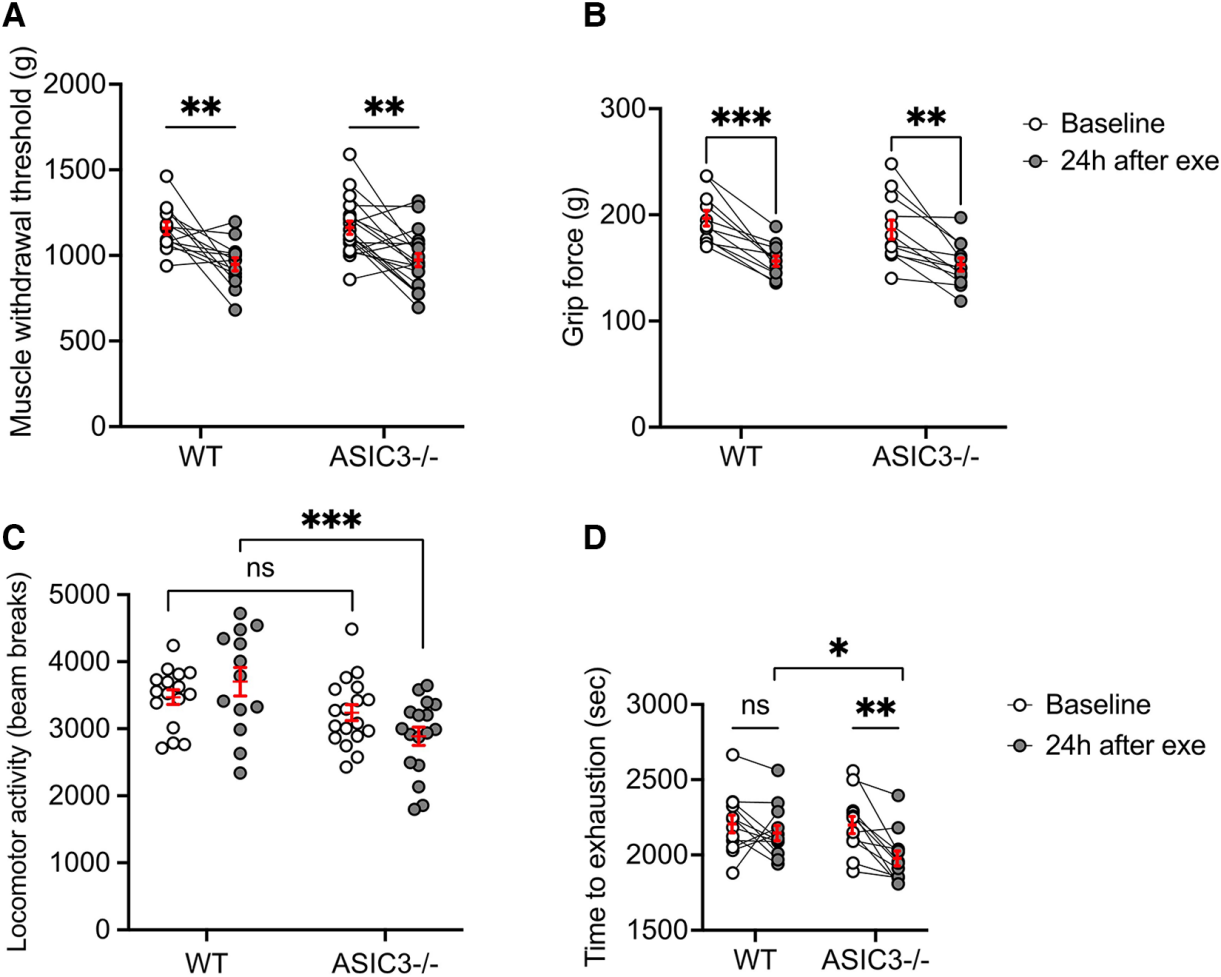
Effect of genetic deletion of ASIC3 on measurements of DOMS. (**A**) MWT was measured at baseline and 24 h after exhaustive exercise in *ASIC3*^−/−^ and WT mice. Two-way repeated measures ANOVA showed a significant time effect [*F*_(1,30)_ = 26.27, *P* < 0.0001, *n* ≥ 13]. Sidak's multiple comparison test revealed both *ASIC3*^−/−^ and WT had a significantly lower MWT 24 h after exercise compared to their baseline (*P* < 0.01). (**B**) Grip strength was assessed at baseline and 24 h after exercise. Two-way repeated measure ANOVA showed a significant time effect [*F*_(1,21)_ = 55.82, *P* < 0.0001, *n* ≥ 11]. Sidak's multiple comparison test revealed both *ASIC3*^−/−^ and WT had a significantly lower grip force 24 h after exercise compared to their baseline (*P* < 0.001 and *P* < 0.0001, respectively). (**C**) Locomotor activity was assessed via open field test at baseline and 24 h after exercise. Two-way ANOVA showed a significant group effect [*F*_(1,60)_ = 13.31, *P* < 0.001, *n* ≥ 13]. Sidak's multiple comparison test revealed significantly lower total beam breaks for *ASIC3*^−/−^ compared to WT mice at 24 h after exercise (*P* < 0.001). (**D**) Exhaustive exercise performance was measured again 24 h after the preliminary exercise test (re-exercise). Two-way repeated-measures ANOVA showed a significant time effect [*F*_(1,22)_ = 12.94, *P* < 0.01, *n* = 12]. Sidak's multiple comparison test found *ASIC3*^−/−^ mice had significantly lower time to exhaustion compared to the baseline (*P* < 0.01) and WT mice at 24 h after exercise (*P* < 0.05).

Since *ASIC3*^−/−^ mice displayed increased fatigue immediately after exercise, this suggested that perhaps ASICs serve as a signal to prevent overexertion and muscle injury. Thus, we hypothesized that *ASIC3*^−/−^ mice might experience altered DOMS compared to WT mice. To test if our exercise protocol induces DOMS in mice, we first tested MWT and grip strength 24 h after exercise. Our results show lower MWT and grip strength 24 h after exercise compared to baseline, indicating the mice developed DOMS, however, the results were not different between WT and *ASIC3*^−/−^ mice (Figures 4A,B). These results suggest that while ASIC3 is required for IEIP, it is not required for DOMS.

Since a hallmark of DOMS is a diminishment of activity involving the same muscle groups, we measured locomotor activity and also measured repeated exhaustive exercise capacity at 24 h after the first exercise test. These tests also involve more volitional movement compared to MWT and grip strength tests. We found that *ASIC3*^−/−^ mice had significantly lower locomotor activity 24 h after exhaustive exercise compared to WT mice (Figure 4C). Additionally, whereas WT mice had a similar exercise performance on repeat treadmill testing done 24 h after the first test, *ASIC3*^−/−^ mice showed significantly reduced maximal exercise time compared to their baseline and compared to WT mice (Figure 4D).

**Figure 5:**
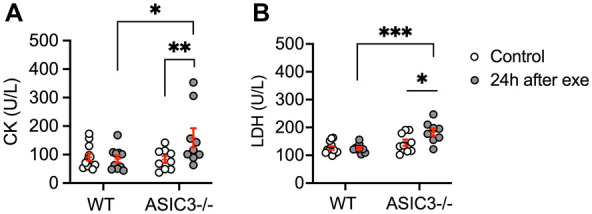
Serum CK (**A**) and LDH (**B**) levels in WT and *ASIC3*^−/−^ mice at baseline (control mice) and 24 h after exercise. (**A**) Two-way ANOVA showed a significant group-time effect [*F*_(1,36)_ = 4.73, *P* < 0.05, *n* ≥ 9]. Sidak's multiple comparison test revealed a significantly higher CK level for *ASIC3*^−/−^ compared to control *ASIC3*^−/−^ (*P* < 0.01) and WT at 24 h after exercise (*P* < 0.05). (**B**) Two-way ANOVA showed a significant group-time effect [*F*_(1,34)_ = 4.73, *P* < 0.05, *n* ≥ 9]. Sidak's multiple comparison test showed a significantly higher LDH level for *ASIC3*^−/−^ after exercise compared to control *ASIC3*^−/−^ (*P* < 0.05) and WT at 24 h (*P* < 0.001).

Lastly, we assessed serum CK and LDH as measures of muscle injury 24 h after exhaustive exercise. We found that *ASIC3*^−/−^ mice had significantly higher levels of CK and LDH compared to WT mice at 24 h after exercise and compared to baseline (Figures 5A,B). In summary, these results show that ASIC3 is not required for DOMS, and in fact, might play a protective role in diminishing DOMS and/or persistent fatigue after exercise.

## 4. Discussion

During strenuous and unaccustomed exercise metabolites and other chemicals accumulate within muscle. Muscle afferents sense these chemical changes, leading to the sensations of muscle pain and fatigue during and immediately after exercise (IEIP), followed a day or so later by DOMS (56, 57). The characteristics and underlying mechanisms of IEIP and DOMS are different. In this study, we show that ASIC3 is required for IEIP but not DOMS. Additionally, since the sensation of fatigue and pain during exercise might protect muscles from overexercising and injury, we tested whether ASIC3 might also play a protective role to prevent muscle injury and attenuating DOMS. We found that compared to WT mice, *ASIC3*^−/−^ mice experienced higher fatigue immediately after exercise. At 24 h after exercise, although both *ASIC3*^−/−^ and WT mice showed similar impairment in strength and muscle pain perception compared to their baseline, *ASIC3*^−/−^ mice showed diminished spontaneous locomotor activity, lower repeat exercise capacity, and increased muscle injury compared to WT mice. Together, these data show that (1) ASICs are required for IEIP but not DOMS, and (2) ASICs might play a protective role against muscle injury and DOMS after exhaustive exercise.

### 4.1. Genetic deletion and pharmacological inhibition of ASIC3 diminished immediate exercise-induced muscle pain (IEIP)

Since high-intensity exercise is associated with the accumulation of protons and other metabolites in skeletal muscle, we hypothesized that ASICs would be required for IEIP. We found that strenuous exercise induces IEIP in WT mice, however, pharmacological inhibition (using APETx2) or genetic deletion of ASIC3 completely abolished mechanical hypersensitivity immediately after exercise. Moreover, we found that buffering pH within skeletal muscle also abolished IEIP, although it should be noted that we did not measure the degree to which our PBS injection prevented acidosis within the muscle interstitium.

The properties of ASICs make them ideal sensors of metabolic changes associated with strenuous exercise, and thus mediators of IEIP. During intense exercise, anaerobic metabolism causes a rapid drop in intracellular pH, which in turn leads to acidosis of the muscle interstitium (pH 6.7–7.0 range) (58). ASICs located at the nerve terminals of muscle afferents are robustly activated within this pH range (59). In addition, lactate and ATP levels are significantly increased within muscle interstitium during intense exercise (60, 61), and both these metabolites are potentiators of ASIC currents (15, 17). If fact, this combination of metabolites may serve to synergistically activate ASICs. Light et al. used calcium imaging to identify a population of muscle afferents that were maximally activated by a combination of protons, ATP, and lactate at physiological concentrations, and pharmacological block of ASICs completely inhibited the response to the combination of agonists (25).

Our results in mice are consistent with recent studies in humans. Stavres et al. found that ASICs blocker (10-mg oral dose of amiloride) significantly improved exercise tolerance under blood flow restriction in healthy adults. Their subjects in the amiloride group performed more work and for a longer period at the same relative level of pain (62). Similarly, Campos et al. reported that amiloride infused into the occluded forearm of human subjects reduced perceived discomfort during metaboreflex activation by postexercise muscle ischemia (63). Additionally, we have previously shown that high-intensity interval training abolished IEIP and was associated with diminished mRNA expression of ASICs in muscle afferents in mice—lending insight into how exercise training can reduce pain (40).

### 4.2. ASIC3 is not required for DOMS

To study DOMS, we tested muscle mechanical hypersensitivity (MWT), strength, spontaneous locomotor activity, re-exercise performance, and chemical measure of muscle injury in *ASIC3*^−/−^ and WT mice 24 h after exhaustive exercise. We found that WT and *ASIC3*^−/−^ mice had similarly lower MWT and grip strength after exhaustive exercise compared to baseline, indicating both groups developed DOMS. Additionally, in other measures, we found that DOMS might actually be worse in *ASIC3*^−/−^ mice compared to WT (discussed in subsequent sections). On the other hand, our mouse *ASIC3*^−/−^ models lacks only the ASIC3 subunit, and the other remaining subunits in muscle afferents (ASIC1a and -2a) still form acid-activated channels (27), so perhaps these subunits contribute to DOMS. Nevertheless, we found no evidence that ASIC3 is required for DOMS.

Our results contradict previous findings. In a rat model of DOMS induced by electrically stimulated contraction of the ankle extensor muscle, either amiloride or the specific ASIC3 inhibitor APETx2 prevented the resultant hyperalgesia as measured by muscle probe withdrawal (41, 42). Moreover, this group recorded from isolated muscle afferent fibers and found them to be hyperexcitable in this same rat model of DOMS, and this hypersensitivity was attenuated by APETx2 (42). Besides the obvious species difference between rat and mouse, what else might account for the differences in our results compared to previous studies examining the role of ASICs in DOMS? One major difference is the mechanism by which DOMS was induced. Whereas we used whole-body high-intensity exercise in conscious mice, previous studies deployed electrically stimulating muscle contraction in anesthetized rats. Additionally, they examined the role of ASICs via pharmacological inhibition, while we used a genetic knockout model of mice (*ASIC3*^−/−^). Finally, previous studies employed only mechanical stimulation to assess DOMS. In addition to mechanical MWT, we more broadly assessed DOMS via spontaneous ambulatory and non-spontaneous ambulatory assessments, muscle force production, and biochemical measures of muscle damage.

Perhaps it should not be surprising that ASICs are not required for DOMS. The accumulation of metabolites begins to wash out of muscle soon after the resolution of exercise and pH, lactic acid and ATP levels are normalized to baseline at the time that DOMS is at its peak (58, 64, 65). It is likely that other receptors, such as TRPV1, that are better poised to sense the inflammatory milieu associated with DOMS are more important in the transduction of muscle soreness in the days after exercise (41, 55, 66, 67). Given the difference in our results compared to previous studies, it is also quite likely that the sensory mechanisms of DOMS vary depending on the type and intensity of muscle activity that induce DOMS, and the stimuli that provoke it during the following 1–2 days.

### 4.3. ASIC3 null mice show increased fatigue immediately after exercise

Since *ASIC3*^−/−^ mice have diminished IEIP, and IEIP can be a barrier to higher exercise performance, we hypothesized that *ASIC3*^−/−^ mice would have higher exercise capacity and run longer on the treadmill. Furthermore, we expected them to experience higher fatigue after exercise due to a higher workload. While we observed no differences in exercise performance between *ASIC3*^−/−^ and WT mice, *ASIC3*^−/−^ mice showed greater fatigue (less spontaneous locomotor activity immediately after exhaustive exercise) compared to WT mice.

To begin to understand these seemingly contradictory results, it is necessary to understand the multiple different roles that ASICs play in exercise. Besides triggering sensations of fatigue and pain, activation of group III/IV muscle afferents during exercise increases blood pressure, heart rate, and ventilation (known as the “exercise pressor reflex”) (29). This reflex is important to increase blood flow to exercising muscle and allow maximal exercise performance. Amann et al. has shown that blocking lower extremity group III/IV muscle afferents during human bicycle exercise (by injection of fentanyl intrathecally into the spinal canal at the lumbar level) reduced feedback from exercising muscle and leads to attenuated blood flow to the leg muscles and faster locomotor muscle fatigue (68–72). The Kaufman group has shown that ASICs are required for components of the exercise pressor reflex (34, 37–39). ASIC3 null rats had a significantly attenuated blood pressure response to metabolite infusion (such as lactic acid) (38). Thus, we speculate that in our study *ASIC3*^−/−^ mice had (1) diminished IEIP, which would tend to increase exercise performance, and (2) an attenuated exercise pressor reflex, which would tend to lower exercise performance. Consequently, we believe that *ASIC3*^−/−^ mice experienced a counterbalance between these two mechanisms which lead to similar exercise performance as WT mice. At the same time, a diminishment of IEIP and the exercise pressor reflex would both be expected to lead to an increase in muscle fatigue in *ASIC3*^−/−^ mice during and immediately following exercise. In addition, while we found equal increases in serum lactate levels in *ASIC3*^−/−^ compared to WT mice, it is also possible that attenuated muscle blood flow in *ASIC3*^−/−^ mice could cause a greater build-up of lactate and other metabolites within exercising muscle, thus contributing to the increase in post-exercise fatigue.

### 4.4. ASIC3 plays a protective role to reduce DOMS and muscle injury

Since ASICs trigger IEIP, and since *ASIC3*^−/−^ mice experienced increased fatigue immediately after exercise, we surmised that sensing metabolites during exhaustive exercise might also serve as an alarm to prevent overexercise and skeletal muscle injury. Thus, we hypothesized that *ASIC3*^−/−^ mice would experience more severe DOMS and delayed recovery after exercise. To test this, we measured DOMS by multiple different techniques. We found no difference between MWT and grip strength tests, whereas *ASIC3*^−/−^ mice showed less spontaneous locomotor activity (open field) and diminished repeat exhaustive treadmill running compared to WT mice measure at 24 h after the initial treadmill testing.

Why the divergent results? A hallmark of DOMS is that the pain is often related to the movement of the same muscle groups involved in the original exercise, thus resulting in a diminishment of activity involving the same muscle groups. However, both MWT and grip strength tests are non-ambulatory tests—MWT does not involve muscle contraction since force is applied by the experimenter, and during grip strength testing the muscle contraction is isometric. On the other hand, spontaneous locomotor activity (open field) is a measure of volitional fatigue/pain. Moreover, both open field and repeat exhaustive treadmill running are tests involving the use and contraction of the same muscle groups as the initial exhaustive exercise, and both are measures of movement-related pain rather than static pain. For these reasons, we believe that measures of spontaneous locomotion and repeat exercise testing might be more discriminative tests of DOMS compared to MWT and grip strength testing. Additionally, we assessed serum CK and LDH as a biochemical measurement of muscle injury and found that *ASIC3*^−/−^ mice had significantly higher levels of CK and LDH compared to WT and their baseline at 24 h after exercise. Thus, these findings suggest that ASIC3 is not only not required for DOMS but may also play a protective function to attenuate DOMS.

In conclusion, these results shed light on the multiple different roles that ASICs play in exercise. We found that although ASIC3 is required for IEIP, it is not required for DOMS and may actually protect against DOMS and muscle injury after high-intensity exercise. These findings have significant implications for health and clinical conditions. Exercise-induced muscle pain can be a barrier to achieving higher exercise performance and can prevent participation in regular physical activity, particularly for people with chronic pain conditions such as fibromyalgia, chronic fatigue, and peripheral artery disease. Understanding the molecular mechanisms of the barriers to exercise can provide a potential target to improve health and fitness.

## Data Availability

The raw data supporting the conclusions of this article will be made available by the authors, without undue reservation.

## References

[1] Campos C Rocha NB Lattari E Paes F Nardi AE Machado S. Exercise-induced neuroprotective effects on neurodegenerative diseases: the key role of trophic factors. Expert Rev Neurother. (2016) 16:723–34. 10.1080/14737175.2016.1179582 27086703

[2] Hamburg NM Balady GJ. Exercise rehabilitation in peripheral artery disease: functional impact and mechanisms of benefits. Circulation. (2011) 123:87–97. 10.1161/CIRCULATIONAHA.109.881888 21200015 PMC3061490

[3] Monda V Villano I Messina A Valenzano A Esposito T Moscatelli F Exercise modifies the gut microbiota with positive health effects. Oxid Med Cell Longev. (2017) 2017:3831972. 10.1155/2017/3831972 28357027 PMC5357536

[4] Polaski AM Phelps AL Kostek MC Szucs KA Kolber BJ. Exercise-induced hypoalgesia: a meta-analysis of exercise dosing for the treatment of chronic pain. PLos One. (2019) 14:e0210418. 10.1371/journal.pone.0210418 30625201 PMC6326521

[5] Hirase T Kataoka H Inokuchi S Nakano J Sakamoto J Okita M. Effects of exercise training combined with increased physical activity to prevent chronic pain in community-dwelling older adults: a preliminary randomized controlled trial. Pain Res Manag. (2018) 2018:2132039. 10.1155/2018/2132039 29849840 PMC5907421

[6] Miles MP Clarkson PM. Exercise-induced muscle pain, soreness, and cramps. J Sports Med Phys Fitness. (1994) 34:203–16. 7830383

[7] Armstrong RB. Mechanisms of exercise-induced delayed onset muscular soreness: a brief review. Med Sci Sports Exerc. (1984) 16:529–38. 6392811

[8] Eston RG Finney S Baker S Baltzopoulos V. Muscle tenderness and peak torque changes after downhill running following a prior bout of isokinetic eccentric exercise. J Sports Sci. (1996) 14:291–9. 10.1080/02640419608727714 8887208

[9] Graven-Nielsen T Arendt-Nielsen L. Induction and assessment of muscle pain, referred pain, and muscular hyperalgesia. Curr Pain Headache Rep. (2003) 7:443–51. 10.1007/s11916-003-0060-y 14604503

[10] Heiss R Lutter C Freiwald J Hoppe MW Grim C Poettgen K Advances in delayed-onset muscle soreness (DOMS) - part II: treatment and prevention. Sportverletz Sportschaden. (2019) 33:21–9. 10.1055/a-0810-3516 30865998

[11] Lieber RL Friden J. Morphologic and mechanical basis of delayed-onset muscle soreness. J Am Acad Orthop Surg. (2002) 10:67–73. 10.5435/00124635-200201000-00009 11809052

[12] Mizumura K Taguchi T. Delayed onset muscle soreness: involvement of neurotrophic factors. J Physiol Sci. (2016) 66:43–52. 10.1007/s12576-015-0397-0 26467448 PMC10716961

[13] Peake JM Neubauer O Della Gatta PA Nosaka K. Muscle damage and inflammation during recovery from exercise. J Appl Physiol. (2017) 122:559–70. 10.1152/japplphysiol.00971.2016 28035017

[14] Allen NJ Attwell D. Modulation of ASIC channels in rat cerebellar Purkinje neurons by ischaemia-related signals. J Physiol. (2002) 543:521–9. 10.1113/jphysiol.2002.020297 12205186 PMC2290513

[15] Birdsong WT Fierro L Williams FG Spelta V Naves LA Knowles M Sensing muscle ischemia: coincident detection of acid and ATP via interplay of two ion channels. Neuron. (2010) 68:739–49. 10.1016/j.neuron.2010.09.029 21092862 PMC3000793

[16] Cadiou H Studer M Jones NG Smith ESJ Ballard A McMahon SB Modulation of acid-sensing ion channel activity by nitric oxide. J Neurosci. (2007) 27:13251. 10.1523/JNEUROSCI.2135-07.2007 18045919 PMC6673420

[17] Immke DC McCleskey EW. Lactate enhances the acid-sensing Na+ channel on ischemia-sensing neurons. Nat Neurosci. (2001) 4:869–70. 10.1038/nn0901-869 11528414

[18] Molliver DC Immke DC Fierro L Paré M Rice FL McCleskey EW. ASIC3, an acid-sensing ion channel, is expressed in metaboreceptive sensory neurons. Mol Pain. (2005) 1:35. 10.1186/1744-8069-1-35 16305749 PMC1308857

[19] Wemmie JA Taugher RJ Kreple CJ. Acid-sensing ion channels in pain and disease. Nat Rev Neurosci. (2013) 14:461–71. 10.1038/nrn3529 23783197 PMC4307015

[20] Kellenberger S Schild L. International Union of Basic and Clinical Pharmacology. XCI. structure, function, and pharmacology of acid-sensing ion channels and the epithelial Na+ channel. Pharmacol Rev. (2015) 67(1):1–35. 10.1124/pr.114.009225 25287517

[21] Lingueglia E. Acid-sensing ion channels in sensory perception. J Biol Chem. (2007) 282:17325–9. 10.1074/jbc.R700011200 17430882

[22] Wemmie JA Price MP Welsh MJ. Acid-sensing ion channels: advances, questions and therapeutic opportunities. Trends Neurosci. (2006) 29:578–86. 10.1016/j.tins.2006.06.014 16891000

[23] Jasti J Furukawa H Gonzales EB Gouaux E. Structure of acid-sensing ion channel 1 at 1.9Å resolution and low pH. Nature. (2007) 449:316–23. 10.1038/nature06163 17882215

[24] Gautam M Benson CJ Ranier JD Light AR Sluka KA. ASICs do not play a role in maintaining hyperalgesia induced by repeated intramuscular acid injections. Pain Res Treat. (2012) 2012:817347. 10.1155/2012/817347 22191025 PMC3236358

[25] Light AR Hughen RW Zhang J Rainier J Liu Z Lee J. Dorsal root ganglion neurons innervating skeletal muscle respond to physiological combinations of protons, ATP, and lactate mediated by ASIC, P2X, and TRPV1. J Neurophysiol. (2008) 100:1184–201. 10.1152/jn.01344.2007 18509077 PMC6195653

[26] Xing J Sinoway L Li J. Differential responses of sensory neurones innervating glycolytic and oxidative muscle to protons and capsaicin. J Physiol. (2008) 586:3245–52. 10.1113/jphysiol.2008.154450 18450773 PMC2538781

[27] Gautam M Benson CJ. Acid-sensing ion channels (ASICs) in mouse skeletal muscle afferents are heteromers composed of ASIC1a, ASIC2, and ASIC3 subunits. FASEB J. (2013) 27:793–802. 10.1096/fj.12-220400 23109675 PMC3545527

[28] Deval E Noël J Gasull X Delaunay A Alloui A Friend V Acid-sensing ion channels in postoperative pain. J Neurosci. (2011) 31:6059–66. 10.1523/JNEUROSCI.5266-10.2011 21508231 PMC6632959

[29] McCloskey DI Mitchell JH. Reflex cardiovascular and respiratory responses originating in exercising muscle. J Physiol. (1972) 224:173–86. 10.1113/jphysiol.1972.sp009887 5039977 PMC1331532

[30] Sluka KA Price MP Breese NM Stucky CL Wemmie JA Welsh MJ. Chronic hyperalgesia induced by repeated acid injections in muscle is abolished by the loss of ASIC3, but not ASIC1. Pain. (2003) 106:229–39. 10.1016/S0304-3959(03)00269-0 14659506

[31] Walder RY Gautam M Wilson SP Benson CJ Sluka KA. Selective targeting of ASIC3 using artificial miRNAs inhibits primary and secondary hyperalgesia after muscle inflammation. Pain. (2011) 152:2348–56. 10.1016/j.pain.2011.06.027 21843914 PMC3476729

[32] Alam M Smirk FH. Observations in man upon a blood pressure raising reflex arising from the voluntary muscles. J Physiol. (1937) 89:372–83. 10.1113/jphysiol.1937.sp003485 16994867 PMC1395054

[33] Kaufman MP Hayes SG. The exercise pressor reflex. Clin Auton Res. (2002) 12:429–39. 10.1007/s10286-002-0059-1 12598947

[34] Ducrocq GP Kim JS Estrada JA Kaufman MP. ASIC1a plays a key role in evoking the metabolic component of the exercise pressor reflex in rats. Am J Physiol Heart Circ Physiol. (2020) 318:H78–h89. 10.1152/ajpheart.00565.2019 31675256 PMC6985806

[35] Ducrocq GP Kim JS Estrada JA Kaufman MP. ASIC1a does not play a role in evoking the metabolic component of the exercise pressor reflex in a rat model of peripheral artery disease. Am J Physiol Heart Circ Physiol. (2020) 319:H171–82. 10.1152/ajpheart.00257.2020 32502377 PMC7474444

[36] Gao Z Henig O Kehoe V Sinoway LI Li J. Vanilloid type 1 receptor and the acid-sensing ion channel mediate acid phosphate activation of muscle afferent nerves in rats. J Appl Physiol. (2006) 100:421–6. 10.1152/japplphysiol.00659.2005 16210435

[37] Hayes SG McCord JL Rainier J Liu Z Kaufman MP. Role played by acid-sensitive ion channels in evoking the exercise pressor reflex. Am J Physiol Heart Circ Physiol. (2008) 295:H1720–5. 10.1152/ajpheart.00623.2008 18723762 PMC2593518

[38] Kim JS Ducrocq GP Kaufman MP. Functional knockout of ASIC3 attenuates the exercise pressor reflex in decerebrated rats with ligated femoral arteries. Am J Physiol Heart Circ Physiol. (2020) 318:H1316–h1324. 10.1152/ajpheart.00137.2020 32302492 PMC7346538

[39] Li J Maile MD Sinoway AN Sinoway LI. Muscle pressor reflex: potential role of vanilloid type 1 receptor and acid-sensing ion channel. J Appl Physiol. (2004) 97:1709–14. 10.1152/japplphysiol.00389.2004 15220301

[40] Khataei T Harding AMS Janahmadi M El-Geneidy M Agha-Alinejad H Rajabi H ASICs are required for immediate exercise-induced muscle pain and are downregulated in sensory neurons by exercise training. J Appl Physiol. (2020) 129:17–26. 10.1152/japplphysiol.00033.2020 32463731

[41] Fujii Y Ozaki N Taguchi T Mizumura K Furukawa K Sugiura Y. TRP channels and ASICs mediate mechanical hyperalgesia in models of inflammatory muscle pain and delayed onset muscle soreness. Pain. (2008) 140:292–304. 10.1016/j.pain.2008.08.013 18834667

[42] Matsubara T Hayashi K Wakatsuki K Abe M Ozaki N Yamanaka A Thin-fibre receptors expressing acid-sensing ion channel 3 contribute to muscular mechanical hypersensitivity after exercise. Eur J Pain. (2019) 23:1801–13. 10.1002/ejp.1454 31314951

[43] Ervilha UF Arendt-Nielsen L Duarte M Graven-Nielsen T. The effect of muscle pain on elbow flexion and coactivation tasks. Exp Brain Res. (2004) 156:174–82. 10.1007/s00221-003-1781-1 14747884

[44] Graven-Nielsen T Svensson P Arendt-Nielsen L. Effects of experimental muscle pain on muscle activity and co-ordination during static and dynamic motor function. Electroencephalogr Clin Neurophysiol. (1997) 105:156–64. 10.1016/S0924-980X(96)96554-6 9152211

[45] Graven-Nielsen T Mense S. The peripheral apparatus of muscle pain: evidence from animal and human studies. Clin J Pain. (2001) 17:2–10. 10.1097/00002508-200103000-00002 11289084

[46] Hodges PW Smeets RJ. Interaction between pain, movement, and physical activity: short-term benefits, long-term consequences, and targets for treatment. Clin J Pain. (2015) 31(2):97–107. 10.1097/AJP.0000000000000098 24709625

[47] Roland MO. A critical review of the evidence for a pain-spasm-pain cycle in spinal disorders. Clin Biomech. (1986) 1:102–9. 10.1016/0268-0033(86)90085-9 23906363

[48] Grundy D. Principles and standards for reporting animal experiments in the journal of physiology and experimental physiology. Exp Physiol. (2015) 100:755–8. 10.1113/EP085299 26076765

[49] Price MP McIlwrath SL Xie J Cheng C Qiao J Tarr DE The DRASIC cation channel contributes to the detection of cutaneous touch and acid stimuli in mice. Neuron. (2001) 32:1071–83. 10.1016/S0896-6273(01)00547-5 11754838

[50] Khataei T Romig-Martin SA Harding AMS Radley JJ Benson CJ. Comparison of murine behavioural and physiological responses after forced exercise by electrical shock versus manual prodding. Exp Physiol. (2021) 106:812–9. 10.1113/EP089117 33527606

[51] Skyba DA Radhakrishnan R Sluka KA. Characterization of a method for measuring primary hyperalgesia of deep somatic tissue. J Pain. (2005) 6:41–7. 10.1016/j.jpain.2004.10.002 15629417

[52] Head SI Bakker AJ Liangas G. EDL and soleus muscles of the C57BL6J/dy2j laminin-alpha 2-deficient dystrophic mouse are not vulnerable to eccentric contractions. Exp Physiol. (2004) 89:531–9. 10.1113/expphysiol.2004.027383 15184359

[53] Jawa A Motara F Moolla M Laher AE. A comparative assessment of the nova stat profile prime plus® critical care analyzer. Cureus. (2020) 12:e9932. 10.7759/cureus.9932 32968593 PMC7505621

[54] Faul F Erdfelder E Lang AG Buchner A. G*power 3: a flexible statistical power analysis program for the social, behavioral, and biomedical sciences. Behav Res Methods. (2007) 39:175–91. 10.3758/BF03193146 17695343

[55] Ota H Katanosaka K Murase S Kashio M Tominaga M Mizumura K. TRPV1 and TRPV4 play pivotal roles in delayed onset muscle soreness. PLoS One. (2013) 8:e65751. 10.1371/journal.pone.0065751 23799042 PMC3684597

[56] Cerqueira É Marinho DA Neiva HP Lourenço O. Inflammatory effects of high and moderate intensity exercise—a systematic review. Front Physiol. (2020) 10:1550. 10.3389/fphys.2019.01550 31992987 PMC6962351

[57] Schranner D Kastenmüller G Schönfelder M Römisch-Margl W Wackerhage H. Metabolite concentration changes in humans after a bout of exercise: a systematic review of exercise metabolomics studies. Sports Med Open. (2020) 6:11. 10.1186/s40798-020-0238-4 32040782 PMC7010904

[58] Bangsbo J Johansen L Graham T Saltin B. Lactate and H+ effluxes from human skeletal muscles during intense, dynamic exercise. J Physiol. (1993) 462:115–33. 10.1113/jphysiol.1993.sp019546 8331579 PMC1175292

[59] Gautam M Benson CJ Sluka KA. Increased response of muscle sensory neurons to decreases in pH after muscle inflammation. Neuroscience. (2010) 170:893–900. 10.1016/j.neuroscience.2010.08.003 20691768 PMC2939292

[60] Cohen RD Woods HF. Lactic acidosis revisited. Diabetes. (1983) 32:181. 10.2337/diab.32.2.181 6337898

[61] Mortensen SP González-Alonso J Bune LT Saltin B Pilegaard H Hellsten Y. ATP-induced vasodilation and purinergic receptors in the human leg: roles of nitric oxide, prostaglandins, and adenosine. Am J Physiol Regul Integr Comp Physiol. (2009) 296:R1140–8. 10.1152/ajpregu.90822.2008 19118095

[62] Stavres J Luck JC Hamaoka T Blaha C Cauffman A Dalton PC A 10-mg dose of amiloride increases time to failure during blood-flow-restricted plantar flexion in healthy adults without influencing blood pressure. Am J Physiol Regul Integr Comp Physiol. (2022) 323:R875–88. 10.1152/ajpregu.00190.2022 36222880 PMC9678418

[63] Campos MO Mansur DE Mattos JD Paiva ACS Videira RLR Macefield VG Acid-sensing ion channels blockade attenuates pressor and sympathetic responses to skeletal muscle metaboreflex activation in humans. J Appl Physiol. (2019) 127:1491–501. 10.1152/japplphysiol.00401.2019 31545154

[64] Li J King NC Sinoway LI. ATP concentrations and muscle tension increase linearly with muscle contraction. J Appl Physiol. (2003) 95:577–83. 10.1152/japplphysiol.00185.2003 12716867

[65] Schwane JA Watrous BG Johnson SR Armstrong RB. Is lactic acid related to delayed-onset muscle soreness? Phys Sportsmed. (1983) 11:124–31. 10.1080/00913847.1983.11708485 27409551

[66] Amaya F Oh-hashi K Naruse Y Iijima N Ueda M Shimosato G Local inflammation increases vanilloid receptor 1 expression within distinct subgroups of DRG neurons. Brain Res. (2003) 963:190–6. 10.1016/S0006-8993(02)03972-0 12560124

[67] Walder RY Radhakrishnan R Loo L Rasmussen LA Mohapatra DP Wilson SP TRPV1 is important for mechanical and heat sensitivity in uninjured animals and development of heat hypersensitivity after muscle inflammation. Pain. (2012) 153:1664–72. 10.1016/j.pain.2012.04.034 22694790 PMC3494878

[68] Amann M Blain GM Proctor LT Sebranek JJ Pegelow DF Dempsey JA. Implications of group III and IV muscle afferents for high-intensity endurance exercise performance in humans. J Physiol. (2011) 589:5299–309. 10.1113/jphysiol.2011.213769 21878520 PMC3225681

[69] Amann M Proctor LT Sebranek JJ Pegelow DF Dempsey JA. Opioid-mediated muscle afferents inhibit central motor drive and limit peripheral muscle fatigue development in humans. J Physiol. (2009) 587:271–83. 10.1113/jphysiol.2008.163303 19015193 PMC2670040

[70] Amann M Venturelli M Ives SJ Morgan DE Gmelch B Witman MA Group III/IV muscle afferents impair limb blood in patients with chronic heart failure. Int J Cardiol. (2014) 174:368–75. 10.1016/j.ijcard.2014.04.157 24794967 PMC4075285

[71] Blain GM Mangum TS Sidhu SK Weavil JC Hureau TJ Jessop JE Group III/IV muscle afferents limit the intramuscular metabolic perturbation during whole body exercise in humans. J Physiol. (2016) 594:5303–15. 10.1113/JP272283 27241818 PMC5023715

[72] Sidhu SK Weavil JC Mangum TS Jessop JE Richardson RS Morgan DE Group III/IV locomotor muscle afferents alter motor cortical and corticospinal excitability and promote central fatigue during cycling exercise. Clin Neurophysiol. (2017) 128:44–55. 10.1016/j.clinph.2016.10.008 27866119 PMC5240388

